# Prevalence and Correlation with Clinical Diseases of *Helicobacter pylori cagA* and *vacA* Genotype among Gastric Patients from Northeast China

**DOI:** 10.1155/2014/142980

**Published:** 2014-05-13

**Authors:** Faisal Aziz, Xin Chen, Xuesong Yang, Qiu Yan

**Affiliations:** ^1^Department of Biochemistry and Molecular Biology, Dalian Medical University, Liaoning Provincial Core Lab of Glycobiology and Glycoengineering, Dalian, China; ^2^Department of Surgery, 2nd Affiliated Hospital of Dalian Medical University, Dalian, China

## Abstract

*Helicobacter pylori vacA* and *cagA* genes have significant genetic heterogenicity, resulting in different clinical outcomes. Northeast part of China has reported high prevalence of *H. pylori* infections and gastric cancer. Hence, we investigated the *H. pylori cagA* and *vacA* genotypes with clinical outcomes in Northeast China. Gastric tissue samples (*n* = 169), chronic gastritis (GIs), gastric ulcer (GU), and gastric cancer (GC) were analysed for 16S rRNA *ureA*, *cagA*, and *cagA* genotypes by PCR. A total of 141 (84%) cases were found positive for *H. pylori* by 16S rRNA and *ureA*. GC showed high *H. pylori* infection (93%) compared with GIs (72%) and GU (84%). The *vacA*s1am1 was highly found in GC (40%) and GU (36%), *vacA*s1am2 in GIs (33%), *vacA*s1bm1 (14%) and *vacA*s1bm2 (8%) in GU cases, and s2m1 in normal cases (33%), while *vacA*s1cm1 showed low frequency in GIs (2%) and GU (3%) and GC showed negative result. The East-Asian *cagA* strain was highly observed in GC (43%), as compared to GIs (41%) and GU (20%). The East-Asian *cagA*/*vacA*s1am1 was significantly higher in GC (23%) than in GU (22%) and GIs (145) patients. The East-Asian type *cagA* with *vacA*s1a and *vacA*m1 is the most predominant genotype in *H. pylori* strains of Northeast China.

## 1. Introduction

*Helicobacter pylori* is a causative agent of gastritis (GIs) and gastric ulcer (GU) diseases, which leads to the development of gastric cancer (GC). Hence it is classified as class I carcinogen by the World Health Organization. Developing countries have* H. pylori* prevalence rate of 80% as compared to developed countries (20–50%) [1, 2]. Genetic diversity may play an important role in genotypic variation of* H. pylori*, which makes it more virulent with diverse pathogenicity [3].* H. pylori *carries different virulence factors, such as urease, flagellar, vacuolating cytotoxin A (VacA), and cytotoxin-associated gene A (CagA), that play an important role in invasion, colonization, and cell proliferation [4, 5]. High genetic variations of* cagA* and* vacA* gene are associated with more severe infectionsof* H. pylori* [6].

VacA toxin encoded by* vacA* gene induces cytoplasmic vacuoles and increases permeability, which leads to the damage of gastric epithelial cells [7]. The* vacA* gene exhibits significant allelic variation in the signal (s) and middle (m) regions. The s-region consists of two major subtypes (s1 and s2) and s1-region has further three subtypes (s1a, s1b, and s1c), whereas m-region designates m1 and m2 subtypes [8, 9]. A pleomorphic combination of s and m regions affects the vacuolating activity of* vacA* gene [10]. Different genotypic combination of* vacA* region results in different pathogenicity level as follows: s1am1 and s1bm1 produce high amount of toxin and are considered the most virulent as compared to s1m2, which produces moderate vacuolating toxins [11, 12]. However, s2m1 and s2m2 are considered less toxic because of their inability to form vacuoles [9]. The s1am1 and s1bm1 subtypes are frequently reported in acute gastritis (GIs), peptic ulcer, and gastric cancer patients, while s2m1 and s2m2 have been reported in the gastric ulcer (GU) patients [9, 13].

Cytotoxin-associated gene A (CagA) is a cytotoxin-associated protein, linked with peptic ulcer and gastric cancer [6].* H. pylori *strains can be grouped as Western and East-Asian subtypes based on polymorphism in 3′ repeat region of the* cagA *gene, which results in the variation of clinical outcome in East-Asian and Western countries. 3′ region of the* cagA* gene consists of two types of repeat regions as 57 bp regions (first repeat region; FR region) and 102 bp regions (Western-type second repeat region; WSR region) [14]. The FR region of East-Asian strains has similar 57 bp region to Western type, but the repeat region of 162 bp was completely different [15]. East-Asian type of* cagA *is more prevalent in East-Asian countries and is more commonly associated with gastric cancer mortality worldwide than Western subtypes [16].

China has high rate of* H. pylori* infections. Some studies reported high prevalence of the* vacA* and* cagA* genotypes from South China. There is little data present regarding the* H. pylori vacA* and* cagA* genotypes and their association with clinical outcomes in Northeast China, where the prevalence of* H. pylori* infection and gastric cancer is much higher than in the south part of China [17]. The* cagA *and* vacA *genotyping is useful to determine molecular epidemiological status of* H. pylori *strain in the northeast part of China. Hence, we aim to evaluate the frequency of* vacA* and* cagA* genotypes with their clinical outcomes in gastric patients from the northeast part of China. This study helps to characterize the more prevalent strain of* H. pylori* in Northeast China, which is useful in diagnosis and treatment of gastric patients.

## 2. Materials and Methods

### 2.1. Patients and Gastroendoscopy

All gastric patients underwent gastroendoscopy and examination at Dalian 1st & 2nd Affiliated Hospital, Dalian, China. Gastric tissues were obtained from the antral and corpus part of the stomach during gastrointestinal endoscopy. Gastric patient's tissue samples were characterized as chronic gastritis (GIs), gastric ulcer (GU), and gastric cancer (GC). Clinical diseases were diagnosed by endoscopic appearance as “normal” in cases of intact mucosa, chronic gastritis, gastric ulcer, and gastric cancer. All the samples were processed and frozen at −20°C until tested. Fresh surgical gastric cancer tissues were collected in transport media and analyzed in laboratory. All gastric samples collected from the patients and the research protocols were in accordance with the Institutional Review Board of Dalian Medical University.

### 2.2. DNA Extraction

The SDS-PK method was adopted to extract DNA from gastric patient's tissues. Gastric tissues were crushed manually and resuspended in 20 *μ*L of 10% SDS, 80 *μ*L of proteinase K buffer (0.5 M EDTA and 4 M NaCl, pH 7.5), 40 *μ*L of proteinase K (10 mg/mL) and make final volume up to 380 *μ*L with sterile water. The mixture was incubated at 55°C overnight. The following day, 100 *μ*L of 6 M NaCl was added, followed by centrifugation at 14,000 rpm for 5 min and the supernatant was separated in new tube. To precipitate the DNA, 500 *μ*L of absolute isopropanol was added, mixed well and centrifuged for 5 minutes at 9000 rpm. DNA pellet was washed with 70% ethanol and air dried. The pellet was resuspended in 50 *μ*L of TE buffer (10 mM Tris and 1 mM EDTA; pH 8.0). Samples were stored at −20°C until used.

### 2.3. Polymerase Chain Reaction (PCR)

PCR was performed to detect* H. pylori *by using specific primers. Target gene, amplicon size, primer names, and sequences are shown in Table 1. For PCR amplification, 1-2 *μ*g of DNA samples was added to a PCR mixture containing 20 pmol forward and reverse primers, 1.5 mM MgCl_2_, 1.5 U of Taq polymerase (Takara, Japan), 2.5 *μ*L PCR buffer, and 200 *μ*M of dNTPs to the total volume of 25 *μ*L. PCR amplification was performed under the following conditions: initial denaturation at 95°C for 3 min followed by 30 cycles of denaturation at 95°C for 30 s, annealing for 30 s (Table 1), polymerization at 72°C for 30 s, and final polymerization at 72°C for 5 min (Bio-Rad Thermocycler). The PCR reaction products were electrophoresed on 1.5% agarose gel with 2000 bp DNA ladder (Takara, Japan) and the bands were visualized by ethidium bromide staining, followed by analysis with Quantity One software (Bio-Rad, USA).* H. pylori* strain was detected by using specific primers targeting 16S rRNA and* ureA *genes (Table 1). The* cagA* and* vacA* statuses were determined from* H. pylori* positive samples by PCR using their respective primers as described in Table 1.

### 2.4. Statistical Analysis

Categorical data were analysed by using chi-square test.* P *value of less than 0.05 was regarded as significant. The statistical software GraphPad prism 5.03 was used for analyzing the data.

## 3. Results

### 3.1. Gastric Patients History

This study was designed to determine the frequency of* cagA* and* vacA* genotypes in gastritis (GIs), gastric ulcer (GU), and gastric cancer (GC) patients from the northeast part of China. A total of 169 gastric patients, 63 males and 106 females ranging in age from 10 to 80 years (average age of 56.32 ± 2.394), were included in the study. Among 169 cases, 51 (30.17%) were diagnosed as GIs, 36 (21.30%) were diagnosed as GU, and 73 (43.19%) samples were regarded as GC, while 9 (5.32%) were normal cases.

### 3.2. Detection of* H. pylori* Infection

*Helicobacter pylori* specific genes (*ureA *and 16S rRNA) revealed that (141) 84% of samples were* H. pylori *positive. These included 6 (54%) normal, 37 (72%) GIs cases, 30 (84%) GU and 68 (93%) of the* H. pylori* positive GC cases (Table 2).

### 3.3. Detection of the* H. pylori vacA* Genotyping and Clinical Manifestations

Among 141* H*.* pylori *positive gastric tissues, we determined six different allelic variants of the* vacA *gene in* H. pylori* positive gastric tissues. Among* H*.* pylori *positive gastric tissues, 51 (36%) were positive for* vacA*s1am1, 24 (17%) for* vacA*s1am2, and 7 (5%) for* vacA*s1bm1, whereas 7 (5%) were* vacA*s1bm2, 2 (2%) were *vacA*s1cm1, and 12 (6%) were positive for* vacA*s2 m1. As shown in Table 2, detailed analysis revealed that 29 (40%) of the GC cases had s1am1 genotype (*P* < 0.05), which was significantly higher than GIs (9) (18%), GU (13) (36%), and normal cases (*P* < 0.05). Respective percentages in GIs, GU, and GC tissue samples of s1am2 were high (33%, 6%, and 6%) as compared to s1mb1 (2%, 14%, and 1%) and s1bm2 (4%, 8%, and 3%)* vacA* genotype. s1cm1 was observed in GIs and GU, while s2m1 was found only in normal and GIs cases (Table 2).

### 3.4. *H. pylori cagA* Genotyping with Clinical Association

We found that the prevalence of* cagA* gene was 86 (61%), out of which 27 (19%) were Western types and 59 (42%) were East-Asian types (Table 2). All negative tissues for 16S rRNA gene were also negative for* cagA *and* vacA *genes. In detailed analysis, Western-type* cagA *gene was observed in 4% of the patients with GIs, 31% with GU, and 18% with GC (*P* < 0.05), whereas 11% of the normal cases were also positive. East-Asian type strains were observed in 41% of the patients with GIs, 20% with GU, and 43% with GC (*P* < 0.05) (Table 2).

### 3.5. Combinational Study of* H. pylori vacA* with* cagA* Genotype

In combinational analysis of* vacA* strain with Western type of* cagA*, as shown in Table 3, we found that 17 (20%) of the* H. pylori* strains have* vacA*s1am1/*cagA *combination, while 7 (8%) of* H. pylori* strains have* vacA*s1am2/*cagA*. In contrast,* vacA*s1bm1/*cagA* and* vacA*s1bm2/*cagA* showed low number of* H. pylori* strains of 2 (2%) and 1 (1%), respectively. The* vacA*s1cm1/*cagA* and* vacA*s2cm1/*cagA* were not found in* H. pylori* positive strains. In detailed analysis, 6 (17%) of GU patients have* vacA*s1am1, 2 (6%)* vacA*s1am2, 1 (3%)* vacA*s1bm1, and 1 (3%)* vacA*s1bm2. Gastric cancer patients showed a number of* H. pylori* strains as follows: 9 (12%) have* vacA*s1am1, 5 (7%)* vacA*s1am2, and 1 (1%)* vacA*s1bm1, and* vacA*s1bm2 has no case reported. GIs patients showed only 1 (2%)* vacA*s1am1 combinational* H. pylori* strain (Table 3). In contrast, combinational analysis of East-Asian type of* cagA* with* vacA* genes demonstrated more frequently* H. pylori* strains of* vacA*s1am1/*cagA* 32 (37%) and* vacA*s1bm2/*cagA* 19 (22%) than* vacA*s1bm1/*cagA* 6 (7%) and* vacA*s1bm2/*cagA* 2 (2%) (*P* < 0.05). However,* vacA*s1cm1/*cagA* and* vacA*s2cm1/cagA were not found in* H. pylori* positive strains. Distribution analysis further showed that GIs patients have 7 (14%) s1am1/*cagA*, 4 (18%)* vacA*s1am2/*cagA,* and 3 (6%)* vacA*s1bm1/*cagA H. pylori* positive strains. In GU patients, we found 8 (22%)* vacA*s1am1/*cagA* and 2 (6%)* vacA*s1am2/*cagA* combinations in* H. pylori *positive strain. In contrast, GC patients showed high number of* vacA*s1am1/*cagA* 17 (23%) and* vacA*s1am2/*cagA* 13 (18%)* H. pylori* positive strains. However, combinations of* vacA*s1bm1/*cagA* and* vacA*s1bm2/*cagA* in GC were found in low percentages as 3 (4%) and 2 (3%), respectively (*P* < 0.05) (Table 3).

## 4. Discussion

Several studies have focused on the diversity of the* H. pylori vacA* and* cagA* virulence genotypes. However, there is little information available related to the frequency of* H. pylori vacA* and* cagA* genotypes in Northeast China. In the present study, we determined the* vacA* and* cagA* statuses among gastritis (GIs), gastric ulcer (GU), and gastric cancer (GC) patients' samples. The prevalence of* H. pylori* was found to be considerably high in the Pacific Asian countries: for example, China has high prevalence of* H. pylori* infection and gastric cancer compared to the rest of the world [23]. In this study, we observed high prevalence of* H. pylori* infection (84%) in gastric patients from 2011 to 2013 (Table 2). Previous studies from the northern and central part of China reported* H. pylori* prevalence rate of 58% from 1990 to 2002 [24]. In comparison, other countries in the same region such as Singapore, Malaysia, Taiwan, and Vietnam showed low* H. pylori *prevalence [23]. These results indicate that* H. pylori* infections have epidemiological diversity, which show different prevalence in different geographical locations.

Genetic variations of the* vacA *genotype make the requirement to investigate clinical outcomes, which is directly linked with the virulence status of* H. pylori*.* vacA* gene shows variation in the signal and midregion that determine* H. pylori* cytotoxin activity. In our study, we predominantly found* vacA* subtypes of s1a, m1, and m2, while s1b and s1c were found in low frequency (Table 2). Our results showed comparable* vacA* frequency rates in China with those from other reported data; for example, Hou et al. reported that s1a and m2 were more prevalent* vacA *genes in China [25], while Mishra et al. found that* vacA*s1a and* vacA *m1 were major subtypes in India [26]. Previously, studies carried out on the Chinese population have reported high prevalence of s1a subtype, while s1b subtype is rarely found in East-Asian countries [27–29]. In another study, s1b subtype was not detected in Hong Kong* vacA* positive* H. pylori* infected patients, while m2 subtype was found in high frequency of 67%. Similarly, we also rarely observed s1c subtype, while s1c was exclusively found from East-Asian isolates [30]. In our study, we found that* vacA*s1am1 (36%) is a more prevalent genotype as compared to the* vacA*s1am2 (17%), while slbm1 (5%), s1bm2 (5%), s1 cm1 (2%), and s2 m1 (6%) genotypes were found in low frequency (Table 2). Previous study showed high prevalence of* vacA *genotype; Ahmad et al. reported the prevalence of s1b/m2 (54.5%) in adult dyspeptic patients from Pakistan [8]. According to Mishra et al.,* vacA*s1a/m1 (53.2%) has predominant genotype in India [26]. However, Hou et al. reported high (90.5%) frequency of* vacA *genotype in China [25].* vacA*s1a and s1b genotypes are prominently associated with high toxin activity and linked to clinical outcome of the diseases. In a detailed analysis of* vacA* genotype, we found high frequency of s1am1 in GU (36%) and GC (40%) patients, while s1am2 was highly found in GIs (33%) and normal cases (11%). s1bm1 and s1bm2 showed high frequency in GU (14%) and (8%), respectively, while s2m1 was found only in normal (33%) and GIs patients (18%) (Table 2). Our results indicate diverse epidemiology of gastritis patients containing* vacA*s2 m1 genotype in the northeast part of China. The* vacA*s1 and* vacA*m1 bearing* H. pylori* strains have been associated with increased virulence capability and higher gastric epithelial damage and ulceration than s2 and m2 strains [31].

*H. pylori* CagA induces pathological alterations, which is closely associated with development of gastritis, gastric ulcer, and gastric cancer.* H. pylori cagA*-positive strains are more virulent causing higher levels of gastric mucosal inflammation in gastritis and gastric cancer [32, 33]. In present study, about 61% of strains were* cagA *positive, which comprises 19% Western-type* cagA* and 42% East-Asian type* cagA* strain. Conversely, our results showed low prevalence of* cagA* gene in gastric patients from Northeast China (Table 2). Previous studies reported high prevalence (93.9%) of* cagA*-positive* H. pylori* strain in China [34–36]. According to Hou et al.,* H. pylori cagA* has high prevalence of 93.2% in Shanghai (the southern part of China) [25]. The neighboring countries of China were also reported to have high prevalence of* cagA*; for example, India has high* cagA* prevalence of 96.2% [26]. Rasheed et al. reported that 52% of* H. pylori *strains carried* cagA *gene with the positivity rate of 80% in GC, 74% in GU, 63% in duodenal ulcer (DU), and 11% in normal cases from Pakistan [37]. Conversely, in Western Europe,* cagA*-positive strains are less prevalent and more frequently found in GU or GC patients [38].

In combinational analysis of* vacA* and* cagA *genotypes, we found that* H. pylori* strains have high frequency of* vacA*s1am1 (37%) or* vacA*s1am2 (22%) with East-Asian type* cagA* genotype (Table 3). Rasheed et al. reported high frequency of* cagA *(61.9%) with predominant* vacA*s1a/m2 genotype in* H. pylori *infected gastric tissues of Pakistani children [37]. A previous study reported significantly higher percentages of* cagA*-positive and* vacA*s1 and* vacA*m1 genotypes with high risk for GC [39]. A recent study conducted in China found no differences in the distribution of* cagA*-positive and* vacA*m genotypes [40]. Our results showed high prevalence of* vacAs1 m1* and East-Asian type* cagA-*positive* H. pylori *strain in gastric cancer patients.

These results might have useful roles in clinical appreciation so as to categorize the specific and most prevalent biomarkers of* H. pylori* strains in the northeast part of China that will be helpful to precisely diagnose large population of gastric cancer patients.* H. pylori *has high capability of developing antibiotic resistance, which make difficult to treat* H. pylori* by conventional antibiotics; however, targeted marker therapy leads to effective* H. pylori* treatment and helps reduce antibiotic resistance.

In conclusion, the present study showed high diversity of the* H. pylori vacA *and* cagA* genotyping. We have reported high frequency of East-Asian type* cagA* with predominant* vacA*s1am1 and* vacA*s1am2 genotypes in gastric ulcer, gastritis, and gastric cancer patients. This study helps to effectively diagnose and treat gastric patients by understanding the trend of* H. pylori* infection in Northeast China.

## Figures and Tables

**Table 1:** Primer sets used for genotyping *H. pylori* by PCR.

Target site	Amplicon size (bp)	Primer names and sequences	Annealing temperature	References
16S rRNA	138	HP-F (5-GCGACCTGCTGGAACATTAC-3) HP-R (5-CGTTAGCTGCATTACTGGAGA-3)	60°C	Gramley et al., 1999 [18]

*UreA *	411	HPU1-F (5-GCCAATGGTAAATTAGTT-3) HPU1-R (5-CTCCTTAATTGTTTTTAC-3)	45°C	Smith et al., 2004 [19]

*vacA*s1a	190	AA1-F (5-GTCAGCATCACACCGCAAC-3) AA1-R (5-TGCTTGAATGCGCCAAAC-3)	56°C	Atherton et al., 1995 [9]

*vacA*s1b	187	SS3-F (5-AGCGCCATACCGCAAGAG-3) SS3-R (5-CTGCTTGAATGCGCCAAAC-3)	56°C	Atherton et al., 1995 [9]

*vacA*s1c	213	S1C-F (5-CTCGCTTTAGTGGGGCTA-3) S1C-R (5-CTGCTTGAATGCGCCAAAC-3)	56°C	Yamaoka et al., 1999 [20]

*vacA*s2	199	SS2-F (5-GCTAACACGCCAAATGATCC-3) SS2-R (5-CTGCTTGAATGCGCCAAAC-3)	56°C	Atherton et al., 1995 [9]

*vacA*m1/m2	570/645	VAG-F (5-CAATCTGTCCAATCAAGCGAG-3) VAG-R (5-GCGTCTAAATAATTCCAAGG-3)	57°C	Yamaoka et al., 1999 [20]

*cagA *	189	CAGA-F (5-TTGACCAACAACCACAAACCGAAG-3) CAGA-R (5-CTTCCCTTAATTGCGAGATTCC-3)	62°C	van Doorn et al., 1998 [21]

Western type *cagA *	Variable	CAGT-F (5-ACCCTAGTCGGTAATGGG-3) CAGW-R (5-TGCCCTACAMCACCSAAACCAC-3) CAGW-F (5-AAAAATTGACCRACTCAATC-3) CAGT-R (5-GCTTTAGCTTCTGAYACYGC-3)	61°C	Yamaoka et al., 1999 [22]

East-Asian type *cagA *	Variable	CAGT-F (5-ACCCTAGTCGGTAATGGG-3) CAGJ-R (5-GCAATTTTGTTAATCCGGTC-3) CAGJ-F (5-GCATCAGCAGGTAAAGGAGT-3) CAGT-R (5-GCTTTAGCTTCTGAYACYGC-3)	52°C	Yamaoka et al., 2000 [14]

**Table 2:** Distribution of *cagA* and *vacA* genotypes with diseases outcomes.

Description	Total *n* = 169		Normal *n* = 9		Chronic gastritis *n* = 51		Gastric ulcer *n* = 36		Gastric cancer *n* = 73	
	*N*	%	*N*	%	*N*	%	*N*	%	*N*	%
*H. pylori* positive	141	84	6	54	37	72	30	84	68	93
*cagA* positive	86	61	1	11	21	41	18	50	46	63
(i) Western type *cagA *	27	19	1	11	2	4	11	31	13	18
(ii) East-Asian type *cagA *	59	42	0	0	21	41	7	20	31	43
*vacAs*1am1	51	36	0	0	9	18	13	36	29	40
*vacAs*1am2	24	17	1	11	17	33	2	6	4	6
*vacAs*1bm1	7	5	0	11	1	2	5	14	1	1
*vacAs*1bm2	7	5	0	0	2	4	3	8	2	3
*vacAs*1cm1	2	2	0	0	1	2	1	3	0	0
*vacAs*2m1	12	6	3	33	9	18	0	0	0	0

**Table 3:** Combinational study between *cagA* and *vacA* genotypes with diseases outcomes.

Description	Total *n* = 169		Normal *n* = 9		Chronic gastritis *n* = 51		Gastric ulcer *n* = 36		Gastric cancer *n* = 73	
	*N*	%	*N*	%	*N*	%	*N*	%	*N*	%
Total *H. pylori* positive	141	84	6	67	37	73	30	83	68	93
Total *cagA* positive	86	61	1	11	21	41	18	50	46	63
Western *cagA/vacA*s1am1	17	20	1	11	1	2	6	17	9	12
Western *cagA/vacA*s1am2	7	8	0	0	0	0	2	6	5	7
Western *cagA/vacA*s1bm1	2	2	0	0	0	0	1	3	1	1
Western *cagA/vacA*s1bm2	1	1	0	0	0	0	1	3	0	0
Western *cagA/vacA*s1cm1	0	0	0	0	0	0	0	0	0	0
Western *cagA/vacA*s2m1	0	0	0	0	0	0	0	0	0	0
East-Asian *cagA/vacA*s1am1	32	37	0	0	7	14	8	22	17	23
East-Asian *cagA/vacA*s1am2	19	22	0	0	4	18	2	6	13	18
East-Asian *cagA/vacA*s1bm1	6	7	0	0	3	6	0	0	3	4
East-Asian *cagA/vacA*s1bm2	2	2	0	0	0	0	0	0	2	3
East-Asian *cagA/vacA*s1cm1	0	0	0	0	0	0	0	0	0	0
East-Asian *cagA/vacA*s2m1	0	0	0	0	0	0	0	0	0	0
